# Development of a model predicting non-satisfaction 1 year after primary total knee replacement in the UK and transportation to Switzerland

**DOI:** 10.1038/s41598-018-21713-2

**Published:** 2018-02-21

**Authors:** Cesar Garriga, Maria T. Sanchez-Santos, Andrew Judge, Thomas Perneger, Didier Hannouche, Anne Lübbeke, Nigel K. Arden

**Affiliations:** 1 Botnar Research Centre, Nuffield Department of Orthopaedics, Rheumatology and Musculoskeletal Sciences, University of Oxford, Oxford, UK; 2 Division of Clinical Epidemiology, Geneva University Hospitals & Faculty of Medicine, University of Geneva, Geneva, Switzerland; 3 Orthopaedic Surgery Service, Geneva University Hospitals & Faculty of Medicine, University of Geneva, Geneva, Switzerland; 4 Arthritis Research UK Centre for Sport, Exercise and Osteoarthritis. Nuffield Department of Orthopaedics, Rheumatology and Musculoskeletal Sciences, University of Oxford, Oxford, UK

## Abstract

We aimed to develop a predictive model for non-satisfaction following primary total knee replacement (TKR) and to assess its transportability to another health care system. Data for model development were obtained from two UK tertiary hospitals. Model transportation data were collected from Geneva University Hospitals in Switzerland. Participants were individuals undergoing primary TKR with non-satisfaction with surgery after one year the outcome of interest. Multiple imputation and logistic regression modelling with bootstrap backward selection were used to identify predictors of outcome. Model performance was assessed by discrimination and calibration. 64 (14.2%) patients in the UK and 157 (19.9%) in Geneva were non-satisfied with their TKR. Predictors in the UK cohort were worse pre-operative pain and function, current smoking, treatment for anxiety and not having been treated with injected corticosteroids (corrected AUC = 0.65). Transportation to the Geneva cohort showed an AUC of 0.55. Importantly, two UK predictors (treated for anxiety, injected corticosteroids) were not predictive in Geneva. A better model fit was obtained when coefficients were re-estimated in the Geneva sample (AUC = 0.64). The model did not perform well when transported to a different country, but improved when it was re-estimated. This emphasises the need to re-validate the model for each setting/country.

## Introduction

Predicting outcomes for chronic disease management represents an important challenge to modern day health systems. The purpose of prognostic investigation is to provide information to physicians to help guide patient management^1^. However, prognostic information is not only used to advise individual judgments but also to make appropriate adjustment when analysing the efficiency of different settings. Here, we present a prognostic model for elective total knee replacement (TKR). Patient satisfaction after TKR correlates with failure of surgery, and non-satisfaction has been related to poorer outcomes after knee replacement^2^. However, the reasons for non-satisfaction are multifactorial. Among them are the patients’ characteristics and expectations, health care provider-related factors, and the health care received^3,4^. Therefore, a framework for what patient features should be taken into account when comparing and predicting patient satisfaction is required. Additional complexity arises when satisfaction is compared between different settings (health care systems), which do not serve the same profile of patients.

Although there are several published studies predicting satisfaction after TKR, often with internal validation^4^, they have not addressed the important issue of transportability^5,6^ of the model, i.e. the ability of the model to function in other countries with a different health care system and/or population^7^. This important step is required to demonstrate usefulness of a model for real clinical practice, to assess whether a single tool can be used worldwide or whether country specific models are required, as demonstrated by the fracture risk assessment tool (FRAX) for predicting osteoporotic fracture^8^. The aim of this study was to develop, validate and assess the transportability of a predictive model for non-satisfaction after primary TKR based on pre-operative factors and surgeon experience.

## Results

### Descriptive statistics

#### Development dataset

We analysed data from 450 (27.9% out of 1616) patients, 64 of whom (14.2%) were non-satisfied with their surgery. Of the 450, 356 (79.1%) patients were operated in Oxford and 94 (20.9%) in Southampton. Mean age and Oxford knee score (OKS) were 70 years (standard deviation (sd): 8 years) and 20 points (sd: 8 points), respectively.

The percentage of missing values in explanatory variables was <10%, except for educational level (16.2%) and surgeon experience (13.1%). We had complete information for sex, age and body mass index (BMI).

#### Validation dataset

Model transportation was carried out on 791 (49.3% out of 1654) patients, 157 of whom (19.9%) were non-satisfied with their operation. Mean age and Western Ontario and McMaster Universities Osteoarthritis Index (WOMAC) were 72 years (sd: 9 years) and 21 points (sd: 8 points), respectively.

Only educational level, WOMAC score and smoking status had missing values for the validation dataset, with 25.0%, 18.8% and 1.3% missing, respectively.

Table 1 shows differences between the UK and Geneva settings. There was a higher proportion of non-satisfied among Geneva patients than UK patients. The Geneva sample had a higher percentage of women, slightly older individuals and more smokers. The UK sample had more obese patients (although this difference was not statistically significant), with more co-morbidities and more treated for depression and knee pain. Educational level, musculoskeletal condition, and proportion treated for anxiety did not differ between the samples. Table 2 presents non-satisfaction events according to candidate predictor category in the UK and the Geneva samples.

**Table 1:** Case mix in UK and Geneva datasets according to candidate predictor category*.

Variable	COASt (sample = 450)		GAR (sample = 791)		*P* value
	n	(%)	n	(%)	
Non-satisfaction	64	(14.2)	157	(19.9)	0.01
Women	262	(58.2)	525	(66.4)	<0.01
Age (mean ± sd years, range)	70 ± 8	28 to 90	72 ± 9	22 to 92	0.01^#^
Higher Education	86/377	(22.8)	147/593	(24.8)	0.48
BMI ≥35Kg/m^2^	101	(22.4)	126	(15.9)	0.14
OKS/WOMAC <25%	98/407	(24.1)	136/642	(21.2)	0.27
MSK condition					0.84
RA-other	55/439	(12.5)	96	(12.1)	
OA	384/439	(87.5)	695	(87.9)	
Co-morbidities					<0.01
0	97	(21.6)	203	(25.7)	
1	118	(26.2)	304	(38.4)	
2	131	(29.1)	197	(24.9)	
3 or more	104	(23.1)	87	(11.0)	
Current smoker	18/414	(4.4)	89/781	(11.4)	<0.01
Treated for anxiety	45/413	(10.9)	77	(9.7)	0.53
Treated for depression	81/410	(19.8)	91	(11.5)	<0.01
Injected corticosteroids	99/408	(24.3)	99	(12.5)	<0.01
Surgeon experience >8 years	179/391	(45.8)	471	(59.5)	<0.01

*Values are the number (%) unless indicated otherwise. ^#^*P* value of Student’s t-test with unequal variance. Clinical outcomes in arthroplasty study, COASt; Geneva arthroplasty registry, GAR; standard deviation, sd; Higher Education = diploma/degree/Doctor of Philosophy; body mass index, BMI; Oxford knee score, OKS; Western Ontario & McMaster Universities Osteoarthritis Index, WOMAC; rheumatoid arthritis, RA; musculoskeletal, MSK; osteoarthritis, OA.

**Table 2:** Non-satisfaction events in UK and Geneva according to candidate predictor category*.

Variable	COASt (sample = 450)		GAR (sample = 791)	
	n (%)	n (%)	n (%)	n (%)
Women/Men	41 (15.6)	23 (12.2)	111 (21.1)	46 (17.3)
Age (mean ± sd years, range)	71 ± 7	50 to 84	69 ± 10	22 to 87
Lower/higher education	42 (14.3)	12 (14.0)	86 (19.3)	24 (16.3)
BMI <35/≥35Kg/m^2^	42 (12.0)	22 (21.8)	129 (19.4)	28 (22.2)
OKS/WOMAC <25%/≥25%	22 (22.5)	39 (12.6)	38 (27.9)	85 (16.8)
RA-other MSK condition/OA	5 (9.1)	56 (14.6)	24 (25.0)	133 (19.1)
0/1 co-morbidity	9 (9.3)	15 (12.7)	47 (23.2)	52 (17.1)
2/3 or more co-morbidities	20 (15.3)	20 (19.2)	42 (21.3)	16 (18.4)
Current/Ex- or non-smoker	5 (27.8)	55 (13.9)	25 (28.1)	130 (18.8)
Treated for anxiety/Non-	12 (26.7)	49 (13.3)	16 (20.8)	141 (19.8)
Treated for depression/Non-	17 (21.0)	41 (12.5)	22 (24.2)	135 (19.3)
Injected corticosteroids/Non-	8 (8.1)	52 (16.8)	19 (19.2)	138 (19.9)
Surgeon experience >8 years/less training	29 (16.2)	26 (12.3)	91 (19.3)	66 (20.6)

*Values are the number (%) unless indicated otherwise. Clinical outcomes in arthroplasty study, COASt; Geneva arthroplasty registry, GAR; standard deviation, sd; Lower education = illiterate, General Certificate of Secondary education, O and A level; Higher Educastion = diploma/degree/Doctor of Philosophy; body mass index, BMI; Oxford knee score, OKS; Western Ontario & McMaster Universities Osteoarthritis Index, WOMAC; rheumatoid arthritis, RA; musculoskeletal, MSK; osteoarthritis, OA.

### Model production and validation

**Figure 1:**
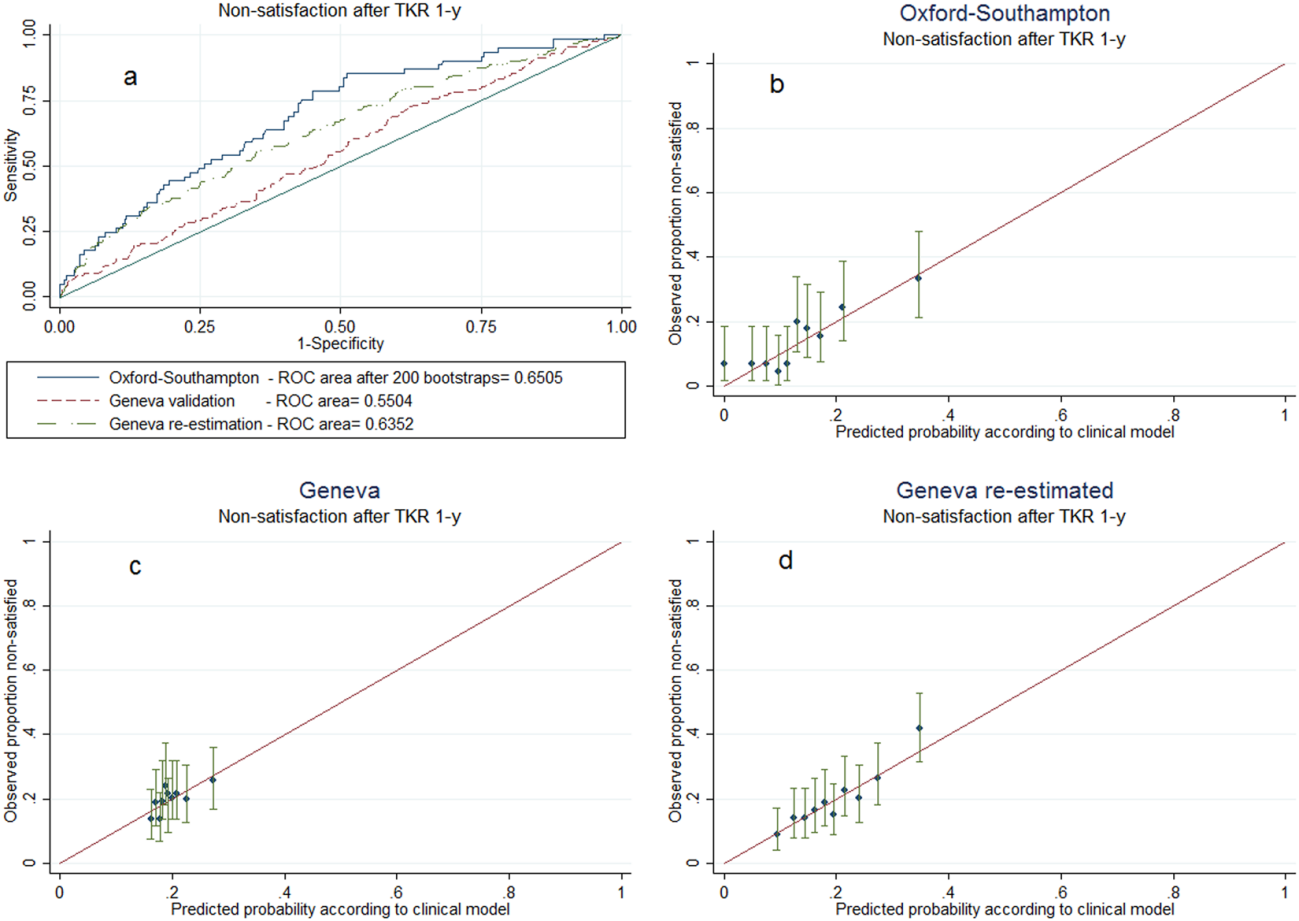
Discrimination and calibration. Upper left panel (a) shows receiver operating characteristic (ROC) curve plots to assess discrimination. Other panels (b,c and d) show the relationship between actual mean score and average predicted probabilities of non-satisfaction, for deciles of risk associated. Upper right panel (b) for the development model, lower left panel (c) for the transported model, lower right panel (d) for the re-estimated model. Bars indicate 95% Agresti–Coull confidence intervals.

In the UK, being treated for anxiety, being current smoker, not having been treated with injected corticosteroids and worse pain and function prior to surgery, were related to non-satisfaction. The logistic regression coefficients with their 95% confidence intervals (95% CI) of the variables selected are summarised in the following equation: non-satisfaction probability = 1/(1 + exp(−(−0.19 × man +0.02 × age at operation +0.997 × prior treatment for anxiety +0.93 × current smoker −1.04 × injection of corticosteroids −0.37 × standardised OKS −3.29))) (Table 3). The model showed moderate discriminatory ability for ascertaining true non-satisfied cases against false non-satisfied cases (Area Under the receiver operating characteristic Curve, AUC = 0.69). Bootstrap validation reduced this to a bias-corrected AUC = 0.65 (Fig. 1).

**Table 3:** Logistic regression models identifying predictors of non-satisfaction 12-month after primary total knee replacement.

Intercept and Predictors (reference category)	COASt (sample = 450)		GAR (sample = 791)	
	Coefficient (95% CI)	*P* value	Re-estimated coefficient (95% CI)	*P* value
Intercept	−3.29		−0.68	
Sex (Women)				
*Men*	−0.19 (−0.82 to 0.44)	0.55	−0.22 (−0.63 to 0.18)	0.28
Age at operation, years	0.02 (−0.01 to 0.06)	0.23	−0.03 (−0.05 to −0.01)	0.003
Prior treatment for anxiety (No)				
*Yes*	0.997 (0.21 to 1.78)	0.01	0.05 (−0.53 to 0.63)	0.86
Current smoker (No)				
*Yes*	0.93 (−0.26 to 2.12)	0.12	0.41 (−0.12 to 0.94)	0.13
Injection of corticosteroids (No)				
*Yes*	−1.04 (−1.87 to −0.21)	0.01	−0.22 (−0.78 to 0.34)	0.45
Standardised OKS/WOMAC, points	−0.37 (−0.67 to −0.07)	0.02	−0.31 (−0.53 to −0.09)	0.006
AUC		**0.69**		**0.64**
Optimism		**0.04**		—
Bias-corrected AUC		**0.65**		—

Clinical outcomes in arthroplasty study, COASt; Geneva arthroplasty registry, GAR; 95% confidence intervals, CI; Oxford knee score, OKS; Western Ontario & McMaster Universities Osteoarthritis Index, WOMAC; Area Under the receiver operating characteristic Curve, AUC.

### Model transportation

Transportation of the model developed in UK to Geneva revealed a lower AUC (0.55) (Fig. 1). Calibration showed good estimation of non-satisfaction but limited to the lower risk estimates (Fig. 1). Finally, when model coefficients were re-estimated using the imputed Geneva sample, an AUC of 0.64 was obtained. The preoperative WOMAC pain and function score was the main predictor (odds ratio (OR): 0.7; 95% CI: 0.6, 0.9). The re-estimated equation for Geneva was: non-satisfaction probability** = **1/(1 + exp(−(−0.22 × man −0.03 × age at operation +0.05 × prior treatment for anxiety +0.41 × current smoker −0.22 × injection of corticosteroids −0.31 × standardised WOMAC −0.68))) (Table 3).

## Discussion

This is the first time a predictive model for non-satisfaction with the outcome of primary TKR for which both internal validation and its ability to be transported to a different country has been assessed. We have demonstrated good internal validation within the UK, but poor transportation to Switzerland when using the *same* model specification. However, re-running the model using the Swiss data to obtain centre specific regression coefficients substantially improved the transportability. Its moderate performance might help to forecast non-satisfaction 1 year after TKR in the UK, but it would require re-estimation in other countries before attempting international use^5^. The model has a moderate predictive capacity to identify non-satisfied patients, therefore, it has limited usefulness to support clinicians and patients in their decisions to undergo a TKR. Further work is still necessary to identify additional risk factors of non-satisfaction to improve discriminatory ability of the model

There are many reasons why an internally validated model may fail to transport to a different country, including a different patient case-mix, different healthcare system (referral patterns, waiting times, and follow-up regimes), surgical training, techniques or implants used.

We found more non-satisfied patients in Geneva, which might be related to the higher proportion of younger patients and of women undergoing TKR in Geneva. In fact, lower mean levels on a visual analogue scale for satisfaction were reported for TKR and unicompartmental knee replacement patients under 55 years of age in an UK hospital^9^. Moreover, women under 60 years of age following TKR were less likely to be satisfied than men in a national (USA), multi-centre study^10^. Women also presented higher non-satisfaction than men in a national survey in Sweden^2^. Smoking could be another potential explanation for the lower satisfaction in Geneva as it was a predictor of outcome and differed substantially in prevalence between the UK and Geneva cohort. The fact that current smokers were less satisfied might be related to stronger residual symptoms 1 year after surgery, higher complications rates, and/or differences in the health care received.

Transportation of the developed model is limited by important differences in BMI and associated co-morbidities. The UK has the highest obesity prevalence in Western Europe and this fact is observed in the comparison of UK and Geneva samples. Obesity is associated with other diseases, thus UK and Geneva patients are not equivalent in terms of comorbidities. Interestingly, there were more non-satisfied patients in the group with no co-morbidities in the Geneva sample, possibly pointing to higher expectations about TKR in younger people who can be expected to have fewer comorbidities.

In the UK sample an injection of a corticosteroid in the months prior to surgery and anxiety treatment were significant predictors while in Geneva they were not. Waiting times for elective TKR are usually one year in the UK as compared to approximately two months in Geneva. Corticosteroid treatment is employed to reduce pain. The shorter waiting time in Geneva may have made the use of this treatment option less frequent.

Other factors to consider in model transportation are unmeasured differences in health care access and socio-economic status. However, in the context of the present study patients, both in the UK and in Switzerland, have universal access to care. Moreover, the proportion of patients with high education was similar. Differences between settings could also be due to post-surgical complications, the number of patients sharing rooms or a negative experience with the staff (i.e. feeling of being treated disrespectfully). Because in this study we restricted the predictor choice to variables known prior to surgery, these factors do not explain poor model transportation here.

Potential differences in non-satisfaction between ethnicities could not be addressed in the model because the vast majority of the patients were white in both UK and Switzerland. Therefore, this model is not generalizable to non-white people for countries where the race is a proxy of socioeconomic status and the access to the health care is not universal^11^. Additionally, the influence of ethnicity on satisfaction is not clear. For example, in the only two studies querying about satisfaction in USA only one found that African-Americans were less likely to be satisfied^12,13^.

It was not possible to compare physical activity levels between UK and Geneva patients. Nonetheless, in the Geneva data physical activity levels did not significantly differ between non-satisfied and satisfied patients, neither before the onset of osteoarthritis (OA) symptoms (6.9, sd: 2.2 vs. 6.6, sd: 2.3; *P* = 0.4) nor prior to TKR (3.6, sd: 1.6 vs. 3.6, sd 1.5; *P* = 0.7). Physical activity was measured using the University of California, Los Angeles (UCLA) Physical Activity Scale, which evaluates level of activity between 1 and 10 (minimum and maximum). Therefore, we would not expect physical activity to be a predictor of non-satisfaction.

To re-estimate the coefficients in the Geneva cohort using the same predictors improved the performance of the model to similar levels as those obtained in the Clinical Outcomes in Arthroplasty study (COASt). This is consistent with the experiences of producing the FRAX tool for predicting fractures in osteoporotic populations, where country specific coefficients were estimated using similar techniques^8^.

Several methodological issues need to be considered. Firstly, the degree of preoperative symptoms (pain and functional disability) were selected as an important predictor of non-satisfaction during the internal validation process. However, different instruments to measure pain and function had been used in the development (OKS) and the validation (WOMAC score) datasets. To address this issue we standardised both scores and observed almost similar proportions of low scores in the validation dataset. Worse preoperative pain and function scores were related to non-satisfaction. High expectations to recover total functionality may be behind this result^4,14^.

Second, greater accuracy but reduced prediction was obtained as a consequence of using bootstrapping to avoid over-fitting. However, transportation to another setting and population further diminished the prediction of the model. Transportation illustrates the difficulty in predicting outcomes in other settings^5,15^. This is because internal validation protects only against over-fitting caused by sampling variation, and not against fundamental differences between populations. A possible solution would be to develop predictive models in multiple setting datasets from the beginning. Then the coefficients would be identical in all settings. Even then the discrimination may vary between settings, e.g. if race was a useful predictor globally this would not help in a racially homogenous setting as ours.

Third, non-satisfaction events in the development dataset were less than a minimum of 100 suggested for developing prediction models using logistic regression^5,16^.

Finally, post-operative factors were not included as previously has been suggested to further improve the prediction of non-satisfaction one year after TKR^17^. This is because, including post-operative factors as confounders, would reduce the chance of finding association between the hospital and the outcome, since the patient’s post-operative status is potentially attributable to the intervention and to hospital care. In addition, we envisage the model to be used in both primary care and pre-operative clinics to assess a patient’s risk of a poor outcome, defined by non-satisfaction, at his/her pre-surgery visit. As such, post-operative parameters would not be available to the clinician or the patient to use the model and help inform the decision making strategy.

We produced and internally validated a model to predict non-satisfaction with outcome after TKR in a UK population. This model did not perform well when transported to a different country, but improved when the model coefficients were re-estimated in the new population. This demonstrated the issues with transporting an internally validated model to a different country, and emphasises the need to re-validate the model for each setting/country.

## Material and Methods

### Source of data and participants

#### Development dataset

The COASt study, is a prospective, dual-centre longitudinal cohort of patients listed for hip and knee surgeries across two UK tertiary hospitals: Southampton University Hospital, and Nuffield Orthopaedic Centre (NOC) in Oxford. Southampton and NOC provide services to some 1.3 million and 655,000 people, respectively. NOC recruited patients between 2010 and 2013. Southampton started recruiting in 2011 and continued to do so in 2015. For this study patients recruited between 2010 and 2014 were included.

#### Validation dataset

The Geneva Arthroplasty Registry (GAR) collects information on socio-demographic variables, comorbidities, medication, PROMs (e.g. WOMAC), radiographs and blood samples (subset) in addition to implant- and surgery-related variables. A prospective longitudinal cohort of TKR patients has been recruited since 1998 at the Division of Orthopaedics and Trauma Surgery of the Geneva University Hospitals. The institution is the only public tertiary hospital in the area serving a population of 500,000 habitants^18^. This analysis included TKRs performed between January 2010 and February 2015. Data from both datasets is available for access to recognised academics. There is a standard application form which must be submitted to a data access committee.

### Inclusion/exclusion criteria

We included patients with OA and rheumatoid arthritis (RA) aged over 18 years and those competent and willing to consent who underwent primary TKR. We excluded from the study those patients with a history of diseases that would be able to mask the outcome analysed, i.e. multiple sclerosis, leg neuropathy, sciatica, stroke or mini stroke, cerebellar ataxia, knee septic arthritis, knee pseudo-gout, avascular necrosis, polymyalgia, systemic lupus erythematous, fibromyalgia, Alzheimer, and poliomyelitis.

#### Development dataset

COASt had 1616 patients undergoing knee replacement: patella-femoral resurfacing (PFR, 16 patients), primary TKR (845 patients), TKR revision (112 patients) and UKR (643 patients). We excluded 107 (6.4%) patients having another disease from the analysis that can mask the outcome. We followed 523 (32.4%) patients who completed and returned the one-year follow-up form. In turn, we excluded 73 (4.5%) patients who did not answer satisfaction question at 1 year on.

#### Validation dataset

GAR contributed with 1654 patients undergoing knee replacement. Specific operations carried out were: primary TKR (1397 patients), TKR revision (115 patients) and UKR (28 patients). 114 (7.1%) patients were excluded because they had a disease meeting the exclusion criteria. Therefore 808 (50.4%) patients who completed and returned the one-year follow-up form were included. In turn, we excluded 16 (1.0%) patients with not response for satisfaction 1 year after TKR.

### Sample size

The development and validation datasets were convenience samples where we included all patients who answered the satisfaction question.

### Outcome: Non-satisfaction

All the patients included in the analysis rated their “overall satisfaction with the outcome of your operation” one year after the surgery. We generated a binary variable grouping satisfied answers (very/somewhat satisfied) versus non-satisfied answers (neither satisfied or dissatisfied, somewhat/very dissatisfied).

### Predictors

Twelve preoperative variables common to COASt and GAR were chosen among those considered relevant by eight surgeons and researchers. Predictors were sex (woman vs. man); age at operation; educational level (higher vs. lower education, i.e. less than university degree); BMI, (<35 vs. ≥35 Kg/m^2^, World Health Organisation (WHO) obesity class II/III); musculoskeletal condition (OA vs. RA), number of comorbidities (liver, bowel, renal, and lung problems, as well as urine infections, diabetes, heart murmur or rheumatic fever, angina or chest pain, heart attack, history of heart failure, pacemaker fitted, history of hypertension, blood clot, unusual bruising or bleeding, and high cholesterol); treated for anxiety; treated for depression; current smoker; intra-articular corticosteroid injection (last 12 months for COASt, injection for OA any time prior to TKA for GAR); surgeon experience (≥8 vs. <8 training years) and; standardised OKS for knee pain and function ((OKS-mean _OKS_)/standard deviation _OKS_, sd). We used standardised WOMAC ((WOMAC-mean _WOMAC_)/sd _WOMAC_) instead of standardised OKS for the validation dataset because OKS was not available for GAR. Lower scores corresponded to most severe symptoms and higher to least symptoms on the standardised OKS and WOMAC scores. To allow the application of the UK score to Geneva patients, both the OKS and WOMAC scores were standardised to a mean of 0 and a standard deviation of 1.

### Statistical analysis

Differences between UK patients and Geneva patients were assessed using χ^2^ test for categorical variables and Student’s t-test for continuous variables.

#### Development and internal validation dataset

A. First, to develop a risk prediction model, we performed the following steps^19^:
  Step 1, imputation of missing values: Multiple imputations on the 12 potential predictors of non-satisfaction were used to address potential bias in the analysis as a result of missing values. Keeping the highest sample size leaded to higher statistical power to predict outcome. 50 imputed datasets were generated using the twelve potential predictors together with the outcome. Imputation also considered the auxiliary variable “hospital where the surgery took place”. Regression coefficients were averaged across the 50 datasets, and standard error was calculated as standard error average plus the variability between the imputations (Rubin’s rules)^20^.
  Step 2, selection of principal predictors: We generated 200 logistic regression models from 200 bootstrap samples. Bootstrapping is a statistic technique that takes randomly patients, with replacement, from the original sample. Some patients may be duplicated, and other patients from the original data may be omitted in a bootstrap sample, being the bootstrap sample size the same as the total number of observations we have in the original sample (n = 450). The aim of this technique is to provide an estimate of the sampling variability with our sample size. For this study, each bootstrap sample was drawn with replacement from the combined 50 imputed datasets. Within each bootstrap sample, the 12 predictors were introduced in a logistic regression model, and an automatic backward selection^21^ was applied using a significance level equal to 0.157, as recommended by Steyerberg^19^. Sex and age were forced into all the models regardless of their *P* value because of their biological relevance^22^.
  Step 3, retention of principal predictors: We retained in the final regression model those variables selected at least 60% of the time. Odds ratio and coefficients with their 95% CI were obtained between each predictor and the outcome using logistic regression.
B. Second, once the principal risk factors were selected, we assessed the performance of the prediction model using discrimination (AUC) and calibration measures. They were represented using calibration and discrimination plots, respectively. Discrimination plot showed the ability of the model to distinguish between non-satisfied patients and satisfied patients. AUC was estimated from the original COASt sample using the final equation obtained (model with selected variables obtained in the previous point). Calibration plots showed the relationship between predicted and observed probabilities of a patient to be non-satisfied. A comparison was done between predictive and observes values for each tenth of predicted risk ensuring 10 equally sized groups. For each decile, the observed proportion of non-satisfied was obtained together with 95% Agresti–Coull confidence interval.
C. Third, to test the internal validity of the model, 200 bootstrap samples with replacement combined with multiple imputations were once again used to evaluate bias-corrected estimates of predictive ability. Bias corrected estimator of AUC was estimated using the following steps: 200 random samples (bootstrap samples) were drawn from the full original sample (imputed COASt dataset of 450 patients). Estimated AUC in each bootstrap model was compared to estimated AUC in the original full sample. Differences in AUC were averaged, providing an average estimated optimism. Subsequently, we subtracted to the overfitted AUC of the imputed COASt dataset the estimated optimism in order to obtain a bias-corrected AUC.

#### Transportability

The transportability of the model was assessed using data from GAR. We generated 50 imputed datasets for GAR using the same potential predictors previously ran in the UK dataset. The equation and the coefficients obtained during the model development were used on GAR dataset to obtain an AUC curve for the Geneva setting. In addition, calibration plot was produced to assess the degree of agreement between observed and predicted probabilities of outcome in GAR sample.

An AUC was also obtained from a model using Geneva data with the same predictors identified in the developed model as sensitive analysis but without the specification of the coefficients. Therefore, the same principal predictors retained for the development model were used to re-estimate new coefficients for the Geneva setting, i.e. a new logistic regression was obtained predicting non-satisfaction for Geneva patients.

All the test used were two-tailed. Analysis were conducted using Stata v.13, and SPSS v.22.

### Ethics

COASt has been approved by the Oxford REC A (Ethics Reference: 10/H0604/91). The sponsoring organisation of the study is the University Hospitals Southampton NHS Foundation Trust (UHS).

The Total Knee Arthroplasty registry prospectively enrolling all patients undergoing knee replacement since 1998. Ethical approval for the registry (No. CER 05-017 (05-041)) was obtained from the Ethical Committee of the Geneva University Hospitals. Data were collected within the two cohorts as confirmed by the study participants in their written informed consent and as directed by the ICH-GCP (International Conference on Harmonisation of Technical Requirements for Registration of Pharmaceuticals for Human Use of Good Clinical Practice) guidelines and appropriate local and international legislation.

The data storage, management and handling was protected and secured in accordance with ICH-GCP guidelines, and with appropriate UK governance regulation (i.e. Data Protection Act, NHS Act 2006, and Health & Social Care Act 2003) and European Commission Directive 95/46/EC.
